# The modulation of auditory novelty processing by working memory load in school age children and adults: a combined behavioral and event-related potential study

**DOI:** 10.1186/1471-2202-11-126

**Published:** 2010-10-07

**Authors:** Philipp Ruhnau, Nicole Wetzel, Andreas Widmann, Erich Schröger

**Affiliations:** 1 Institute of Psychology I, University of Leipzig, Seeburgstr. 14-20, D-04103 Leipzig, Germany

## Abstract

**Background:**

We investigated the processing of task-irrelevant and unexpected novel sounds and its modulation by working-memory load in children aged 9-10 and in adults. Environmental sounds (novels) were embedded amongst frequently presented standard sounds in an auditory-visual distraction paradigm. Each sound was followed by a visual target. In two conditions, participants evaluated the position of a visual stimulus (0-back, low load) or compared the position of the current stimulus with the one two trials before (2-back, high load). Processing of novel sounds were measured with reaction times, hit rates and the auditory event-related brain potentials (ERPs) Mismatch Negativity (MMN), P3a, Reorienting Negativity (RON) and visual P3b.

**Results:**

In both memory load conditions novels impaired task performance in adults whereas they improved performance in children. Auditory ERPs reflect age-related differences in the time-window of the MMN as children showed a positive ERP deflection to novels whereas adults lack an MMN. The attention switch towards the task irrelevant novel (reflected by P3a) was comparable between the age groups. Adults showed more efficient reallocation of attention (reflected by RON) under load condition than children. Finally, the P3b elicited by the visual target stimuli was reduced in both age groups when the preceding sound was a novel.

**Conclusion:**

Our results give new insights in the development of novelty processing as they (1) reveal that task-irrelevant novel sounds can result in contrary effects on the performance in a visual primary task in children and adults, (2) show a positive ERP deflection to novels rather than an MMN in children, and (3) reveal effects of auditory novels on visual target processing.

## Background

For successful adaptive behavior, we should be able to focus attention on information being relevant in a given context (voluntary attention), but at the same time should remain responsive to perturbing events in order not to dismiss potentially important information (involuntary attention). An optimal balance between the processes subserving voluntary and involuntary attention is a prerequisite for the development of many cognitive functions. This balance obviously depends on contextual factors such as the demands imposed by the difficulty of a given task and it may vary with age. Our study investigated the processing of task-irrelevant but attention catching novel sounds in children and adults and its modulation by working memory load. We expect that distraction of attention and the processing of subsequent target stimuli are affected by novels differently between age groups. Working memory load is expected to affect novelty processing in both age groups, with possible differences in children deriving from the still immature cognitive control system.

Attentional capture elicited by unexpected new or changed sounds and its consequences on behavioral performance in visual and auditory tasks have been extensively studied in auditory-visual distraction paradigms in adults (for a review see [1]). Usually, subjects are instructed to discriminate by a button press between two categories of visual stimuli (e.g. odd and even numbers) presented in a random order at a constant rate (e.g. one stimulus in 1.2 s). A task-irrelevant sound occurs shortly (e.g. 300 ms) before each visual stimulus. The sound is either a frequently occurring sinusoidal standard tone or a novel sound drawn from a pool of environmental sounds. When subtract novel-related ERPs from standard-related ERPs the so-called novelty complex consisting of N1/Mismatch Negativity (MMN), reflecting automatic deviancy detection, P3a, reflecting an involuntary attention switch to the deviation, and Reorienting Negativity (RON), reflecting the re-allocation of attention to the primary task, can be observed [1-11]. Additionally, a prolongation of reaction times (RTs) and/or a decrease of hit rates in the visual discrimination task are observed (e.g. [2-9,11]). Similar ERP and behavioral effects are obtained in an analogous auditory-auditory distraction paradigm (e.g. [1,10-16]).

In recent years some developmental studies could show a similar ERP complex in children of different ages using environmental novel sounds [17-20] or sinusoidal deviants [21-23]. Although the underlying functions are still immature - apparent in larger distraction effects (RTs to deviant minus standard trials) compared to adults [24] - it seems as if children process deviations quite similar to adults [25]. Recently Wetzel and colleagues [20] could show that children at the age of 7-8 years are partly able to control auditory distraction voluntarily, indicated by lower distraction effects and a decrease of RON amplitude when novel sounds are predictable, even though they not yet achieve the level of adults.

The processing of novel or deviant sounds and the effect it exerts on the performance in a primary task is modulated by task demands [12,26-29]. However, the findings about the influence of working memory load on distraction are not consistent. Lavie [30] and Muller-Gass and Schröger [27] show that high load increases distractibility whereas Berti and Schröger [12] and San Miguel and colleagues [28] report reduced distraction under high memory load. It seems possible that channel separation between task-relevant information and task-irrelevant distracting information has an interactive effect with task demands in determining the extent of auditory distraction. Namely, with increasing load distraction increases if channel separation is possible, e.g. when target and distractor are presented in different modalities (as in [28]), and distraction decreases if channels separation fails, e.g. when target and distractor are on the same object (as in [27]).

**Figure 1:**
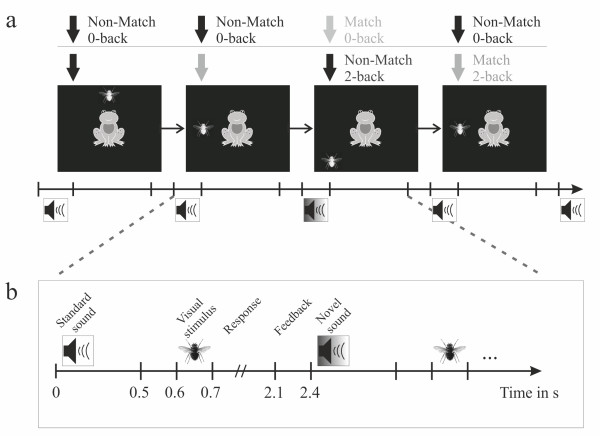
**Experimental paradigm**. (a) Subjects had to decide whether the fly occurred at a predefined position (0-back = low load) - one of the four corners of the screen - or whether the stimulus of the current trial occurred at the same position than the stimulus two trials before (2-back = high load). Before each visual stimulus a task irrelevant standard (88%) or a novel sound (12%) was presented. (b) In the displayed box the time line of one trial is shown.

There are still no developmental studies combining memory load and distraction research by using ERPs. To our knowledge this is the first study which investigates modulation of novel processing by working memory load in children combining behavioral and electrophysiological measures. We developed an auditory-visual paradigm (see Figure 1) ensuring a distinct separation of task-relevant and task-irrelevant, presumably perturbating information following the footprints of San Miguel and colleagues [28]. Working memory is needed to fulfill cognitive tasks and therefore keeps available a small amount of information in a readily accessible state [31]. Hence it is important to know the characteristics of this "small amount", in other words the capacity of working memory. There is a broad literature investigating working memory development and its estimation in children [32-35]). One main finding is that working memory capacity is increasing intensively during school years [31,34]. This makes it difficult to manipulate working memory load in quantitatively identical steps in different age groups. Therefore, the present study used a qualitative change in altering demands on working memory. Manipulation of memory load via n-back tasks has been turned out as a successful approach in children [36]. In our study, subjects had the task to either evaluate the position of a visual stimulus (0-back, low load) or to compare the position of the current stimulus with the one two trials before (2-back, high load). This is more a qualitative manipulation as no memory for previous stimulus positions is required for the 0-back task while it is for the 2-back task. The experiment was furthermore designed as a game to increase and keep the motivation of the children at a high level.

We expected that novels elicit a P3a and RON in children and adults, with children showing a preceding positive MMR [20,37,38]. According to previous research [12,28,39] task load was not expected to influence the MMR but should affect later steps of novelty processing reflected by P3a and RON in adults [12,27,28]. Whether it affects novelty processing in children was an open question. As children cannot inhibit task-irrelevant information as effectively as adults [20], it can be expected that children show high distractibility with novel sounds, which increases with memory load [27]. On the other hand -if the results of Berti and Schröger [12] or San Miguel and colleagues [28] apply - it may also be the case that distraction decreases with increasing load. However, considering again immature inhibitory processes [20], this should be found to a lesser extent in children than in adults.

Few studies along this line looked for ERP effects of auditory distractors on the processing of subsequent visual stimuli. Escera et al. [3] found a reduced visual N1 amplitude in trials preceded by a novel sound, whereas San Miguel et al. [29] found that novels in the auditory domain led to an enhancement of the visual ERP to subsequent visual targets in the P3b range (300-400 ms post stimulus). In the latter study the P3b effect was accompanied by a facilitation of reaction times to novels compared with standard sounds. The authors assume that novels might serve as alerting signal reflected by the facilitation and the P3b enhancement [29].

Unlike previous studies utilizing the auditory-visual distraction paradigm, we chose a relatively long stimulus-onset-asynchrony (SOA) of 600 ms between sound and visual stimulus. This might obscure behavioral distraction effects (which have been shown to decrease with increasing SOA [4,40]) but has the advantage that auditory and visual ERPs can be disentangled to a larger extent. According to the study by San Miguel and colleagues [29], we expected an effect of the auditory novel to the visual P3b, elicited by targets, in adults and tested for a similar effect in children.

## Results

### Reaction times and hit rate

Table 1 contains the average reaction times and hit rates for both age-groups (children aged 9-10 years vs. adults aged 18-33 years) in both memory load conditions. RTs were prolonged in the high-load condition relative to the low-load condition in children and adults. Adults had a RT prolongation (distraction effect) in trials comprising a novel while children had a RT reduction (facilitation effect) in novel trials compared to standard trials. The repeated measures ANOVA for within-subject factors Memory Load (high vs. low load), Stimulus Type (standard vs. novel sound) and the between subject factor Age (adults vs. children) revealed a main effect of Memory Load (*F*(1,24) = 73.209, *p *< 0.001, *η*^2 ^= 0.753) indicating slower RTs in high load condition compared to low load condition. Further an interaction of Stimulus Type × Age (*F(1,24) *= 17.113, *p *< 0.001, *η*^2 ^= 0.416) was obtained, confirming distraction effects in adults (RT novels > RT standards, *t(11) *= -3.364, *p *< 0.01) and facilitation effects in children (RT novels < RT standards, *t(13) *= 2.885, *p *< 0.05). The equivalent ANOVA was applied for the hit rates (HRs) and revealed a main effect of Memory Load (*F(1,24) *= 41.432, *p *< 0.001, *η*^2 ^= 0.633) indicating lower hit rates in the high load condition (87.12%) relative to the low load condition (97.26%) and a main effect of age (*F(1,24) *= 5.163, *p *< 0.04, *η*^2 ^= 0.177) indicating lower hit rates in children (90.34%) relative to adults (94.35%). No interaction was observed (all *F(1,24) <*1.7, *p *> 0.2).

**Table 1:** Mean reaction times (RT) and hit rates (HR) with standard errors of the mean (SEM).

		Children (N = 14)				Adults (N = 12)			
			
		RT(ms)	SEM	HR(%)	SEM	RT(ms)	SEM	HR(%)	SEM
**Low Load**	**Standard**	439	18.71	96	0.84	375	12.26	99	0.20
	**Novel**	422	12.98	96	0.77	399	11.92	99	0.29
		
**High Load**	**Standard**	599	35.66	84	2.05	565	28.76	89	2.41
	**Novel**	568	36.24	86	2.33	580	28.92	90	2.77

### Auditory ERPs

**Figure 2:**
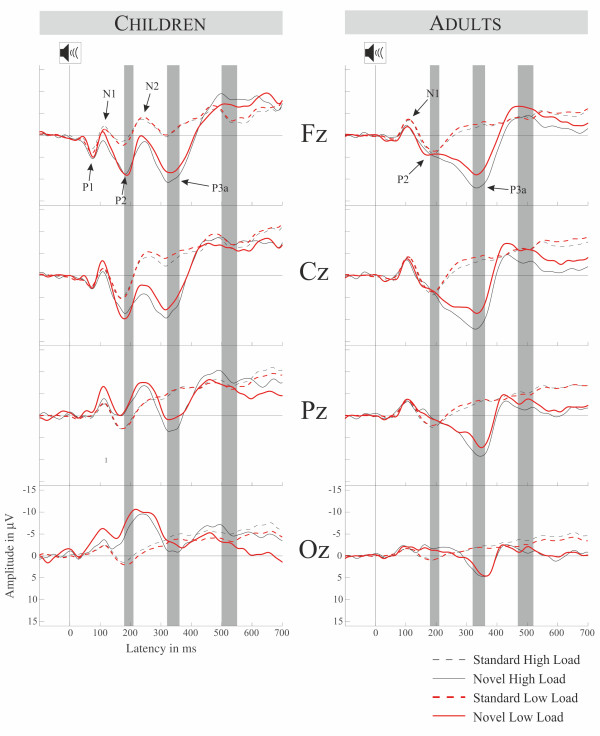
**Group average ERPs in the memory load conditions for both age groups**. Left panel displays children right panel displays adults with age typical event related responses. The high lightened areas mark the statistically analyzed areas of MMR, P3a and RON.

**Figure 3:**
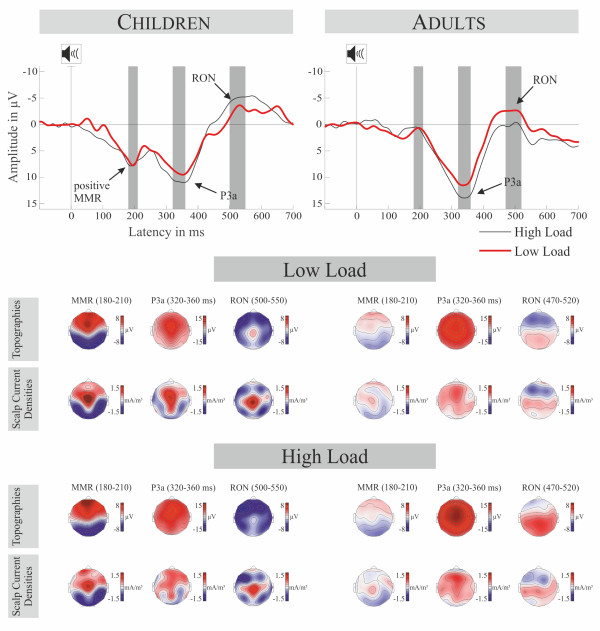
**Group average difference waves and scalp topographies for the time windows of MMR, P3a and RON**. Upper panel: Difference waves (novel minus standard sound) for both age groups at Fz. Time windows for the statistical analysis are highlighted. Lower Panels: Voltage and scalp current density (SCD) topographies for the time windows of MMR, P3a and RON in both memory load conditions for both age groups (children left, adults right).

Auditory ERPs are displayed at Figure 2, their novel minus standard difference waves, difference topographies and corresponding SCDs are displayed at Figure 3. The ANOVA computed for the N1 refractoriness effects (peak 100 ± 10 ms) in adults revealed no significant main effect or interactions, most importantly the Stimulus Type main effect was not significant (*F(1,11) *= 2.059, *p *> 0.17) though there was no different N1 on novels compared to standard sounds.

In the MMN time-window (peak 190 ± 10 ms), there was an evident novelty-related positivity (MMR), with a posterior negativity in children without a corresponding effect in adults. The corresponding ANOVA for the MMN time-window with factors Memory Load, Stimulus Type and Age revealed a main effect of Stimulus Type (*F(1,24) *= 15.662, *p *< 0.002, *η^2 ^*= 0.395) and a Stimulus Type × Age interaction (*F(1,24) *= 9.726, *p *< 0.006, *η^2 ^*= 0.288). T-tests did not show a significant MMN for adults (*t(11) *= 0.693, *p *> 0.5) but confirmed a positive MMR for children (*t(13) *= 5.105, *p *< 0.001).

Both age-groups showed a characteristic P3a which was confirmed in the ANOVA of the amplitudes in the P3a-window (peak: 340 ± 20 ms) revealing a main effects of Stimulus Type (*F(1,24) *= 74.802, *p *< 0.001, *η^2 ^*= 0.757). It also revealed a main effect of Memory Load (*F(1,24) *= 6.566, *p *< 0.02, *η^2 ^*= 0.215) indicating more positive ERPs for standards and novels in the high memory load condition, but no interaction between Stimulus Type and Memory Load (*F(1,24) *= 2.31, *p *> 0.15). The ANOVA of the peak latencies of the P3a with the factors Memory Load and Age revealed no significant effects (all *F(1,24) *< 1.61, p > 0.22). The equivalent ANOVA of the amplitudes in the RON time-window (adults: peak 495 ± 25; children: peak 525 ± 25) revealed a main effect of Stimulus Type (*F(1,24) = *6.611, *p *< 0.02, *η^2 ^*= 0.216) indicating the presence of the RON and a significant three-way interaction Stimulus Type × Memory Load × Age (*F(1,24) *= 6.462, *p *< 0.05, *η*^2 ^= 0.212). This interaction is elucidated by two additional 2 × 2 ANOVAs with factors Stimulus Type and Memory Load separated for the age groups. In adults, there was an interaction of Stimulus Type × Memory Load (*F(1,11) *= 10.167, *p *< 0.01, *η^2 ^*= 0.480) indicating an influence of load on ERPs elicited by novels (high load vs. low load, *t(11) = *3.003, *p *< 0.05) but not on ERPs elicited by standard sounds (high load vs. low load, *t(11) *= -0.560, *p *> 0.5) and thus a more negative RON in the high load condition. On the other hand, children showed no Stimulus Type × Memory Load interaction (*F(1,13) = *1.853, *p *> 0.19) and thereby no effect of memory load on the RON. The ANOVA of the peak latencies of the RON with the factors Memory Load and Age revealed a main effect of Age (*F(1,24) *= 43.57, *p *< 0.001, *η^2 ^*= 0.654) confirming a later RON in children compared to adults. No other effects were observed.

### Visual ERPs

**Figure 4:**
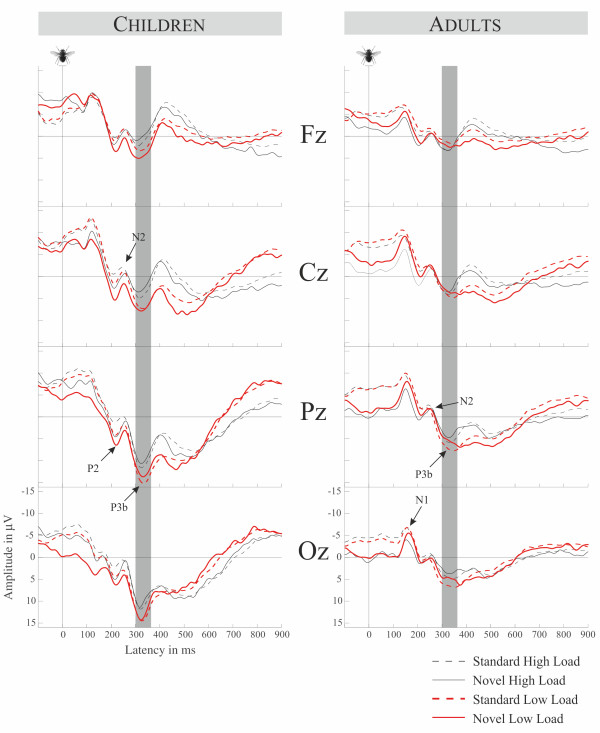
**Group average ERPs in the memory load conditions for both age groups time-locked to the visual target stimulus**. Left panel displays children right panel displays adults. The P3b analysis time window is highlighted.

The ERPs time locked to the onset of the visual stimulus following novel respectively standard sounds are shown in Figure 4. The visual ERPs in adults show a distinct N1 (200 ms after onset of the visual stimulus) followed by a small P2 and N2 and a distinct parieto-occipital P3b. In the standard and novel ERPs for the 2-back condition, an overlapping frontal negativity can be seen subsequent to P3b. Children also revealed an N1 which was followed by P2 and N2 deflections. The subsequent P3b to all stimulus types and conditions and the fronto-central negativity to standards and novels in the 2-back condition can also be identified. The ANOVA in the P3b time-window (330 ± 30 ms) with the factors Memory Load × Stimulus Type × Age revealed no interactions but a main effect of Memory Load (*F(1,24) = *12.633, *p *< 0.003, *η*2 = 0.345), confirming less positive P3b in the high load condition. Furthermore there was a main effect of Stimulus Type (*F(1,24) = *10.062, *p *< 0.005, *η^2 ^= *0.295), confirming less positive P3b amplitudes in trials containing novels compared to standards, and a main effect of Age (*F(1,24) = *10.273, *p <*0.005, *η^2 ^= *0.300) explained by higher amplitudes in children compared to adults.

## Discussion

For the present study we developed a paradigm useful to examine distraction and memory load in a developmental context. Our results indicate that the initial novelty processing of children differs from adults (MMR), that orienting towards the novel is relatively similar between children and adults (P3a) but that reorienting mechanisms are not yet fully developed (RON). On the behavioral level novels surprisingly speeded the children's response whereas they slowed the response in adults.

### Behavioral Data

Both age groups responded faster and more accurate in the low load condition compared to the high load condition. This indicates that the manipulation of the working memory load was successful in both age groups. However, the memory load manipulation revealed no age effects. As expected, children generally responded less accurate than adults.

Novel sounds caused behavioral distraction in adults as reflected by increased reaction times to the visual target when preceded by a task irrelevant novel compared to when preceded by a standard sounds. Similar effects of task-irrelevant novel or deviant sounds on task performance were shown before (e.g. [1,14]). These effects could not necessarily be expected, as with increasing SOA between sound onset and onset of the target-related information behavioral distraction events caused by deviant sounds get smaller [4,40]. Most interesting, in contrast to adults, children showed a facilitation effect, that is, responses to visual targets were speeded when the preceding sound was novel. Such a facilitation effect has been reported in adults [41,42]. San Miguel and colleagues [41] proposed that (together with other factors) attentional task demands and the temporal position of the novel relative to the encoding or retrieval of the task-related visual information influences whether a novel causes distraction or facilitation. Our study shows that the impact of a novel sound on the performance in a visual primary task is different between school children and adults. Within the theoretical framework of San Miguel and colleagues, we conclude that the alerting potential of novels is larger in children than in adults (at least in the present paradigm). Children do reveal a distraction effect with shorter SOAs between distractor and target information in auditory-visual [18,43] and auditory-auditory distraction paradigms [19,21,24,25]. Thus, it is possible that it takes some time for the facilitation effect to develop.

A different explanation for facilitation in adults comes from a very recent work by Parmentier et al. [42]. They consider novelty distraction to be modulated by the informational value of the sound. If both task irrelevant sound types, standard and novel, contain the same information about the foreground task (for example about the timing a target will appear), novels cause distraction on behavioral level. But if the novel sounds but not the standard sounds contain information they even result in facilitation. Considering the smaller sensory memory in children [44] and the large SOA we chose, it may be possible that children could not carry the informational value of the standard sounds to the primary task. The novels, due to their activating nature, may have delivered the timing information more efficiently resulting in a shortage of the reaction time. On the other hand, and in line with Parmentier et al. [42] adults could have picked up the target information from both stimulus types and thus get distracted from the violation of the auditory pattern.

In the present study, the novelty effect was not modulated by task load (although task load had a clear effect on RTs per se). This adds to the divergent findings with respect to the effect of task load on distraction. It seems that depending on various factors - such as the specific nature of the task or the SOA - an increase in task-load can increase or decrease distraction or it can have no effect.

From a developmental point of view our results are quite astonishing as usually distractors should impair behavior, especially in children. It should be the purpose of future studies to elaborate how this facilitation evolves and which aspects are responsible for it.

### Auditory ERPs

The auditory ERPs per se show morphologic differences between children and adults which are consistent with previous studies (e.g. [19,21,45,46]). However, the P3a appears to be relatively similar between the age groups. Thus, although sensory processing, at least its neural basis reflected by the ERPs, is still immature in children, the novel-specific attentional orienting as reflected by P3a is already well developed at the age of 9-10. This is consistent with recent findings about distraction and cognitive control [20]. However, mechanisms underlying RON seem to operate differently in children and adults.

The difference waves of novels and standard sounds in the present paradigm resulted in a positive mismatch response in school children aged 9-10, which previously was only reported in kindergarten children, infants [19,38,47], and children aged 7-8 years [20]. Voltage and SCD topographies for this MMR point to a complex generator structure. A prominent central source is accompanied by fronto-lateral sources and parieto-temporal sinks. A combination of temporal areas and deeper central sources appears plausible. Neural sources of the MMR in temporal areas are in line with source modeling done by Maurer and colleagues [38]. Importantly, this MMR was absent in adults and they did also not show an MMN to novels. This absence of MMN was expected on the basis of previous studies showing that MMN is difficult to obtain with omissions of stimulus features [20,48]. Considering that the environmental sound we used as a standard had a broad frequency spectrum, a novel sound consisted in the omission of parts of the frequency spectrum of the standard. In other words, our stimuli compensate for novelty effects due to different refractory states of novel and standard. We accomplished to control for these effects as was confirmed by our analysis of the N1 time window. These N1 refractoriness effects may in fact be main contributors to the MMN with novels (see [49] for a review). As such, our results could be seen as further evidence for this interpretation (see also [20] for similar results). It is still unclear which characteristics are responsible for the elicitation of a positive MMR compared to an MMN. Considering that no other novelty-specific ERP is elicited before the MMR and its latency-similarity to the MMN in adults, it is quite convincing that the MMR reflects the change detection mechanism in the novelty complex [1]. Furthermore, Maurer and colleagues [38] could show that the MMR is not related to attentional orienting, but is sensitive to the same experimental manipulations as the MMN. However, our results clearly indicate a difference in how novels are processed in children and adults in the time-window of 180-200 ms.

It is still not known which parameters lead to the elicitation of the MMR and, furthermore, its underlying mechanism still has to be investigated intensively. Wetzel et al. [19] proposed that the long SOA between the sounds is responsible for the frontal positivity. They interpret that the still immature prefrontal cortices lead to a different stimulus processing visible in this positivity. On the other hand, Ĉeponienė et al. [50] varied the SOA in 7-9 year old children and found neither an attenuation nor amplification of the MMN. But unlike the study of Wetzel et al. [19], using novels as distractors, Ĉeponienė et al. [50] used deviant sinusoidal tones. So it appears that the SOA alone is not sufficient to elicit this positivity. It may well be that complexity of the sounds play a more important role.

Supporting this idea, some studies report a quite similar positivity with a latency in the MMN time-range in children ages 9-13 years [17,18,37], which is referred to as early P3a (eP3a) circumscribed from the late P3a, also detailed investigated in adults (for a review see [26]). All mentioned developmental studies used novel sounds and an SOA of at least 1.7 s. The topography of the eP3a is fronto-central and quite focal as is the MMN, only with inversed polarity. Also its generator is thought to be in superior temporal areas, basically the auditory cortex [17] corresponding well to eP3a generators found in adults [51,52]. Thus, the positive deflection in our data may be the described eP3a. In the terms of Ĉeponienė and colleagues [37] it may be that the eP3a governs the attentional shift which is reflected by the late P3a. It is argued that the eP3a is somehow an "attentional-domain 'receiver'", calling for the attentional switch (lP3a) [37]. This is further corroborated by the latency difference of early and late P3a - of around 100 ms which is of the same order needed to shift attention from one spatial focus to another [32]. Also the decreased latency difference in the eP3a-lP3a complex in children compared to adults [52] coincide with general latency decreases of ERP components found from child- to adulthood [46]. Hence, the similarities of the topographies and scalp current densities of the eP3a in children (see Figure 3 and [18]) and adults (see [26]), especially the central source, support the interpretation of both as the same process.

Besides MMR and eP3a there is a third way of how the presented positivity can be interpreted. As its latency is in the P2 range it may be argued that the effect is a modulation of the P2 (see Figure 2). Also the central source in the MMR time window, visible in the SCDs in Figure 3, is supporting this notion. Unfortunately it is even harder to speculate on the underlying function when we regard the MMR as a P2 effect, because the functions underlying the P2 are almost as widely circumscribed as its generators (for a review see [53]). However, a recent article argued that the P2 mechanism serves to efficiently regulate access to perceptual representations [54]. In this framework our results may indicate that this mechanism in children needs much more effort to access the representation of novels compared to standards, another hint for children being more stimulus driven than adults. In other words, adults may access the perceptual representation of novels and standards similarly, and process the deviating aspect of novels differently thereafter (P3a and RON). In children novels and standards may yield different perceptual representations reflected in the different P2 amplitudes.

Of course we may also think of the MMR as a conglomerate of the described processes including mismatch detection, attention shift government and access to perceptual representations. It is crucial to further explore this positivity in future studies to disentangle its underlying functions and contributing sources.

However, we could show that MMR with a positive polarity at fronto-central leads are not confined to newborns or kindergarten children but can be obtained with children aged 9-10, which show a classical MMN of negative polarity with smaller SOAs and sinusoidal tones instead of novels [20,21].

Voltage topographies of the P3a show that children's P3a has a more focused, central distribution, whereas the P3a of adults is broader (but also with a central maximum). This is also confirmed by the SCD topographies which show a different pattern of sinks and sources in children, with one central source and two parietal sinks, than in adults, showing only one fronto-central source. Statistics in the P3a interval showed no effect of age on the processing of novels. That leads to the assumption that the mechanism of orienting on task irrelevant stimuli is already well mature at the age of 9-10 years. In that same interval, we showed larger amplitudes in the high load condition in both age groups. This indicates that memory load modulates the brain activity following the task irrelevant sounds. However, this modulation was statistically not dependent on standard or novel sound presentation, i.e. it did not matter whether the sound was violating a pattern or not. So memory load appears to be not mediating the attention switch on task irrelevant novel sounds.

It has been shown, that the P3a is sensitive to working memory load manipulation [12,27,28] indicating a different amount of attentional resources oriented on the distracting stimulus under different load conditions. An explanation of our data is to be found in the structure of our task. In this cross-modal design subjects may have been able to focus efficiently on the visual task. Thereby distractor and task stimulus may have been processed in parallel or at least independently. Evidence for this hypothesis comes from behavioral data by Parmentier et al. [55]. These authors propose that attention capture and target processing are independent as long as they are perceptually distinct, which would be the case in our design. Another possibility is delivered by Muller-Gass and Schröger [27] who show that the amount of attentional resources determine the extent of distraction. In their study stimuli were carrying both, task relevant and distracting information, increasing the amount of attentional resources on the distractor. In our case attentional resources are directed away from the distractor, leaving the same amount of resources in both load conditions for the orienting process reflected by the P3a. A third distinctive feature of our design is the long sound-target SOA of 600 ms. It seems possible that task load did not modulate the attention shift towards the irrelevant sound (reflected by P3a), because the time between the sound and the visual task relevant stimulus was too long. However the novelty complex is clearly affected by load in the time window following the P3a, which will now be discussed.

In the RON time window scalp voltages show the typical frontal pattern in adults, also confirmed by two frontal sinks in the SCDs. In children however, SCDs show additionally to the frontal sinks a prominent central source and two parietal sinks. Additionally, and in contrast to the P3a, the RON was delayed in children compared to adults. This extends the finding reported by Horváth and colleagues [21] for 6 year old children to a different age group. Furthermore it strengthens the idea of a functional independence of P3a and RON. Future studies should investigate the development of this delay from early to late school age. Contradictory to the MMR and P3a, RON is clearly affected by the memory load manipulation in adults whereas it is not in children. This results support recent findings [20] showing that children 7-8 years old are more stimulus-driven than adults. While in the present study in adults the reorienting process is diminished under high memory load, it elicits the same response in children independently of the current load. The findings in adults also support results by Berti and Schröger [12], who found a decreasing RON with increasing memory load. Following their argumentation we can assume that adults were able to highly focus on the demanding task thereby decreasing the resources admitted to involuntary attention switches, here re-orienting of attention to the task. It was shown before that these executive functions within working memory, which are necessary for the balance between voluntary and involuntary attention resources, are less developed in children especially when it comes to highly salient distractors as used in the present paradigm. As the RON in the children's group is similar in both load conditions we assume that the underlying process of reallocation of attention to the task is carried out to the same extent.

Our divergent results in the P3a and RON time windows are confirmed by recent studies which questioned the strength of the link between P3a and RON in adults [56-58]. Furthermore, comparing children aged 7-8 and adults Wetzel and colleagues [20] also find dissociations between P3a and RON. Together with our results this indicates that the underlying mechanisms of P3a and RON are not only partly independent but also developing at different ages. It appears that the involuntary attention shift is already well developed around 9 - 10 years while the reorientation mechanism is not.

### Visual ERPs

So far there are only few studies investigating the modulation of visual ERPs by auditory novelty. Escera et al. [3] found reduced visual N1 and San Miguel et al. [41] increased visual P3b following auditory novels. We found that visual target detection reflected by P3b (e.g. [59]) is impaired by novels, independently of memory load. This effect was present for adults and children. There are different explanations for this effect. It is likely that the processing of the auditory novel captures attentional resources that are missing at the later processing of the visual stimuli. Recently Parmentier and colleagues confirmed in a behavioral cross-modal distraction study the hypothesis that the cognitive locus of distraction originated in the shifts of attention occurring between attention capture by the novel sound and the onset of the visual target processing [55]. According to a model of distraction in the cross-modal oddball task by Parmentier and colleagues [55], the influence of the auditory novels and the visual P3b could also be boosted by a semantic effect triggered by the novelty processing. In other words, the cognitive system is (to some extent) still engaged in novelty processing.

## Conclusion

The present cross-modal distraction study yielded different initial processing of novel sounds in children and adults (reflected by MMR), whereas the following attention switch was similar in both age groups (reflected by P3a). Subsequently, reorienting of attention after distraction was affected by memory load in adults but not in children (reflected by RON), suggesting that adults, unlike children, are able to restrict the attentional resources to the novelty processing if the task is highly demanding. At the behavioral level novels yielded distraction in adults but facilitated the performance in children. We assume that the alerting aspect of novels exceeds the distracting aspect only in children. Finally, novel sounds modulated the processing of the visual target stimuli at the ERP and the behavioral level.

## Methods

### Subjects

Twelve adults (mean age: 24; 2 [years; month], range: 18; 11-33; 4, 6 female, 10 right handed) and 16 children (mean age: 10; 4, range: 9; 4-10; 11, 7 female, 13 right handed), participated in the present study. Handedness was measured with an adapted German version of the Oldfield Scale [60]. Two children did not complete the experiment after the training block due to poor performance (hit rate < 40% and 56%). They did not continue in order to prevent frustration. The remaining subjects (especially the children) liked to play the game. Subjects gave their written (resp. the parents of the children) and oral consent. All subjects reported being healthy, having normal hearing and a normal or corrected to normal sight. Adults were paid for their participation and children received a voucher for toys, CDs, DVDs, books etc. of a local store. The study was approved by the local ethical committee of the Medical Faculty of the University of Leipzig and was conducted following the code of Ethics of the World Medical Association (Declaration of Helsinki).

### Stimuli

A frog (3.4 × 4° visual angle) was presented centrally as fixation point (Figure 1). Visual target stimulus was a 2.2 × 1.8° big fly, which was presented at an eccentricity of 4.2° visual angle at one out of eight positions around the frog. Auditory stimuli consisted of a buzzing mosquito sound with 500 ms duration as standard and 52 environmental novel sounds with 500 ms duration, which were rated as identifiable in a previous pilot study. Novels could only occur once in the same condition (see below). Sounds were presented with an intensity of 68 dB (measured by HMS III, head acoustics, Herzogenrath, Germany) through a speaker on each side of the subject, at a distance of 0.9 m and in a 45° angle.

### Procedure

Participants were seated in an acoustically and electrically shielded chamber. The experimental stimulation was presented using Matlab 7.1 (Mathworks Natick, MA) in combination with the Cogent Graphics toolbox (developed by John Romaya at the LON at the Wellcome Department of Imaging Neuroscience). Task load was manipulated using a visual n-back task. In the high load condition (2-back) subjects had to memorize the position of a fly (8 possible positions), which occurred every 2.4 s, and compare it to the position of the fly two trials before (see Figure 1 for the paradigm). In a forced choice task subjects had to press one response button if it was the same position (match) and another button if the positions were different (non-match). Responses had to be given using the index fingers. In the low load condition subjects had to press one response button if the fly appeared on a predefined position (one of the four corners of the screen) and another button when it appeared at any of the other positions. The target positions were restricted to the corners around the frog. Their order was pseudo randomized within subjects to prevent sequence effects. Sides of the buttons were counterbalanced across subjects throughout the experiment. The reaction time window was limited to 1.4 s after onset of the visual stimuli according to studies using a similar paradigm [32,36]. The response was followed by a feedback (smiling or sad looking frog) indicating whether the response was right or wrong. Every visual target stimulus was preceded by either a frequent standard sound or a rare novel sound. SOA from auditory to visual stimulus was 600 ms. Subjects were instructed to ignore the sounds and concentrate only on the visual stimulation. Before preparing the electrodes, all children completed a training of the high load condition. To prevent frustration and securing a task load manipulation the experiment was conducted only with participants who achieved hit rates exceeding 70% in the training. The whole experiment consisted of 8 blocks (4 in the high and 4 in the low load condition), each of 4.5 minutes length. Each block consisted of 105 trials containing in the visual stimulation 32 (30.5%) matches and 73 (69.5%) non-matches of the visual targets, and 13 (12.4%) novels and 92 (87.6%) standard sounds. The whole experiment, including preparation, breaks, and removal of the electrodes and hair washing had a maximum duration of 2 hours.

### Behavioral and electrophysiological recordings and data analysis

Reaction times, hit rates and EEG were measured. Only responses between 200 and 1200 ms were included in the analysis. EEG was recorded with BioSemi amplifiers (BioSemi, Amsterdam) at a digitization rate of 512 Hz. Electrodes (Ag/Ag-Cl-electrodes) were placed at the following positions: Fp1, AF3, F7, F3, FC1, FC5, T7, C3, CP1, CP5, P7, P3, Pz, PO3, O1, Oz, O2, PO4, P4, P8, CP6, CP2, C4, T8, FC6, FC2, F4, F8, AF4, Fp2, Fz, Cz and at the left (M1) and right (M2) mastoids. Raw data processing was performed with the EEGLAB toolbox by Delorme and Makeig [61]. EOG was measured using the setup described by Schlögl et al. [62], with one electrode at nasion and two electrodes on the outer canthi. Following Schlögl et al. [62] an automatic eye-movement-correction was applied on the data, preceded by a 0.5 to 100 Hz offline band-pass filter (FIR, Filter-order = 3072, Kaiser-window). The corrected signal was filtered offline with a low-pass filter of 30 Hz (FIR, Filter-order = 188, Kaiser-window) and all electrodes were re-referenced to the right mastoid. On individual level epochs from -100 ms to 1500 ms were computed and averaged for the two different stimulus types in the two memory load conditions. Epochs of -200 ms to 800 ms with amplitude change exceeding 150 μV were discarded from the analysis. A pre- stimulus window of 100 ms served as baseline. Epochs containing standards following epochs containing novel sounds were not included in the analysis, as standards following deviating stimuli may elicit a mismatch response themselves [63]. Voltage and scalp current density (SCD) topographies were computed for the novel-minus-standard difference waves in the both memory load conditions. SCDs were computed as the second spatial derivative of the spherical spline interpolated potential distribution [64,65]. The maximum degree of the Legendre polynomials was chosen to be 50, and the order of splines (m) was set to 4. A smoothing parameter lambda of 1e-7 was applied.

### Statistical analysis

Behavioral and EEG data were statistically analyzed using repeated measurements ANOVAs. For the behavioral data and the auditory deviance-related ERP component amplitudes (MMN/MMR, P3a and RON) an ANOVA including within-subject factors Memory Load (high load vs. low load), Stimulus Type (standard vs. novel) and the between subject factor Age (9-10 vs. 18-33 years) was applied on Fz. To test for N1 refractoriness effects ANOVAs in the adult group were computed, as children do not show a corresponding N1 [45]. This analysis contained the factors Memory Load and Stimulus Type and was performed on Fz. To test for age related latency effects individual peak latencies for P3a and RON were identified in the difference waves (novel minus standard sounds) on Fz and tested with an ANOVA with the factors Memory Load and Age. In participants who showed ambiguous peak patterns the peak closest to the average peak was measured. Effects on the processing of the visual stimuli were analyzed using an ANOVA with the factors Memory Load, Stimulus Type and Age in the time window of visual P3b. This analysis was performed on Pz, where P3b usually has its maximum (see [66], for a review). Greenhouse-Geisser-correction was applied where appropriate. Statistically significant ANOVA results were further analyzed with paired *t*-tests. For all statistical tests an alpha level of .05 was defined. As effect size partial eta square is reported.

## Authors' contributions

PR, NW and ES designed the study. AW and PR programmed the task. PR acquired the data. AW and PR performed the data analysis. All authors participated the data evaluation and interpretation and in writing the manuscript, and have approved the final version of the manuscript.
